# Experiences of childhood, intimate partner, non-partner, and hate crime-related violence among a sample of people living with HIV in the epicenter of the U.S. HIV epidemic

**DOI:** 10.3389/fpubh.2024.1331855

**Published:** 2024-02-07

**Authors:** Jessica M. Sales, Katherine M. Anderson, Melvin D. Livingston, Sophia Garbarino, Selaem Hadera, Eve S. Rose, Madelyn S. Carlson, Ameeta S. Kalokhe

**Affiliations:** ^1^Department of Behavioral, Social, and Health Education Sciences, Emory University Rollins School of Public Health, Atlanta, GA, United States; ^2^Department of Medicine, Emory University School of Medicine, Atlanta, GA, United States; ^3^Hubert Department of Global Health, Emory University Rollins School of Public Health, Atlanta, GA, United States

**Keywords:** interpersonal violence, ACES, intimate partner violence, hate crimes, people living with HIV

## Abstract

**Introduction:**

Experiences of violence among people living with HIV (PLWH) are thought to be highly prevalent but remain inadequately captured. As a first step toward acceptable, trauma informed practices that improve engagement and retention in care for PLWH, we must acquire more comprehensive understanding of violence experiences. We examined experiences of various forms of lifetime violence: adverse childhood experiences (ACES), intimate partner violence (IPV), non-partner violence (NPV), and hate crimes among diverse sample of PLWH in Atlanta, Georgia.

**Methods:**

Cross sectional data collected from in- and out-of-care PLWH (*N* = 285) receiving care/support from Ryan White Clinics (RWCs), AIDS Service Organizations (ASOs), or large safety-net hospital, February 2021–December 2022. As part of larger study, participants completed interviewer-administered survey and reported on experiences of violence, both lifetime and past year. Participant characteristics and select HIV-related variables were collected to further describe the sample. Univariate and bivariate analyses assessed participant characteristics across types of violence.

**Results:**

High prevalence of past violence experiences across all types (ACES: 100%, IPV: 88.7%, NPV: 97.5%, lifetime hate crimes 93.2%). People assigned male at birth who identified as men experienced more violence than women, with exception of non-partner forced sex. Participants identifying as gay men were more likely to have experienced violence.

**Conclusion:**

Among our sample of PLWH at the epicenter of the United States HIV epidemic, histories of interpersonal and community violence are common. Findings emphasize need for RWCs, ASOs, and hospital systems to be universally trained in trauma-informed approaches and have integrated onsite mental health and social support services.

## 1 Introduction

Violence is increasingly recognized as a major public health problem. The spectrum of types of violence individuals encounter is vast, including adverse childhood experiences (ACEs), intimate partner violence (IPV), community-based violence (i.e., non-partner physical and sexual assault, hate crimes, gang violence), terrorism and war/combat. Though no individual is immune to violence, violence is disproportionately experienced by certain populations. For instance, while experienced by 1 in 7 United States (US) children (1). ACEs are disproportionately reported by individuals residing in Black, low-income, and urban communities (2–5). Maguire-Jack et al. (3) found that among a national child sample, Black children were significantly more likely to have ACE exposures than white children, with 64% of Black children having at least one ACE exposure compared to 41% of white children (3). Another national study found that compared to all other income groups, people with annual incomes lower than $15,000 had significantly higher ACE exposure (4). Additionally, hate crimes are more common among sexual and gender minority (SGM) populations (6, 7); an estimated 20% of SGM Americans have experienced a hate crime (8). Flores et al. (6) found that lesbian, gay, bisexual, and transgender (LGBT) individuals are nearly 9 times more likely to experience a violent hate crime than non-LGBT people. Sexual violence and intimate partner violence are higher among women than men;- compared to one in 13 men, nearly one in five women report contact sexual violence in the US (9). Forty-seven percent (47%) of women versus 44% of men in the US report lifetime contact sexual violence, physical violence, and/or stalking by an intimate partner (9). Overall, exposure to violence varies significantly by race, ethnicity, sexual orientation, gender identification, geographic location, and more (8).

There are over 1.2 million people living with HIV (PLWH) in the US, with over 36,000 new diagnoses in 2021 alone (10). HIV is disproportionally prevalent among historically under-resourced groups, including men who have sex with men (MSM), Latinx individuals, and Black women (10). In 2021, MSM and Latinx individuals accounted for 67 and 29% of all HIV diagnoses, respectively, while Black women accounted for 57% of HIV diagnoses among females (10). HIV increasingly impacts trans individuals, with diagnoses rising 74% among trans men and 21% among trans women between 2017 and 2021 (10). These HIV disparities are largely driven by homophobia, racism, stigma, and poverty (10). Perhaps unsurprisingly given who is most burdened by HIV in the US and the intersecting identities that many people with HIV hold (e.g., being female, being Black; being gay, being Latinx), PLWH also report experiencing violence at rates greater than those without HIV. For example, intimate partner violence is experienced by 68–95% of cisgender women with HIV (11), 68–77% of cisgender men with HIV, and 93% of transgender PLWH (11–13). A small qualitative study suggests that MSM with HIV may experience intimate partner violence at even higher rates than women with HIV (12). Specific to experiences of childhood violence, Henny et al. found that 53 and 39% of PLWH experienced childhood physical and sexual abuse, respectively (14), and another study found that reports of childhood physical and sexual abuse were significantly higher among women with HIV compared to those without HIV (15). Non-partner violence is also disproportionately experienced by those with HIV, as illustrated by a study among female sex workers which found that those with HIV were nearly four times more likely to experience physical and sexual violence than those without HIV (16).

As an upstream determinant of health, violence has direct effects on HIV care engagement and HIV viral suppression. It also has indirect effects through mental health and substance abuse pathways (17). Therefore, violence screening and intervention could be a critical first step in reducing mental health disorders, substance use disorders (SUDs), and downstream HIV-associated morbidity and mortality. Violence is often considered a traumatic experience. As defined by the American Psychiatric Association, a traumatic experience involves a threat to one’s physical or emotional well-being, and elicits intense feelings of helplessness, terror or lack of control (18). Such experiences can alter a person’s perception of themselves (e.g., self-efficacy), their environment (e.g., reaction to clinic environment and medical procedures), and the people around them (e.g., trust in providers) (18). The downstream negative effects of violent traumas, particularly IPV, are apparent on mental health (i.e., depression, post-traumatic stress disorder (PTSD), SUDs) (19–21), and HIV-related outcomes (i.e., CD4, viral load, opportunistic infections, AIDS mortality) (22–25).

Unfortunately, our understanding of the impact of violence on HIV outcomes and care engagement is largely limited to IPV experiences of heterosexual women or childhood experiences of violence (11, 26–31). Relatively little is known about the experiences of PLWH across multiple types of violence, the impact of other types of violence on HIV care continuum outcomes, and if and how experiences of violence differ by sex and gender. Acquiring a more comprehensive understanding of experiences of violence among both males and females with HIV, holding multiple intersecting identities, is a necessary first step to help tailor and prioritize violence screening and intervention practices for PLWH to address violence as a critical but often unaddressed social determinant of health. Thus, to begin filling this gap we examined experiences of various forms of lifetime interpersonal and community violence in a diverse sample of PLWH in the Southern US.

## 2 Materials and methods

### 2.1 Study overview

As part of a larger quantitative study of violence experiences among PLWH and their impact on HIV care outcomes, between February 2021–December 2022 we conducted a cross-sectional survey among PLWH (*N* = 285) in Atlanta, Georgia to assess the comprehensive experiences of various forms of lifetime interpersonal and community violence.

### 2.2 Study setting

The epicenter of the US HIV epidemic is in the southeastern region (10). The state of Georgia has the highest rate of new infections and over half of those infections occur in the Atlanta metropolitan area (32). This region experiences high poverty, unemployment, racism, transphobia/homophobia, and poor access to healthcare (33) and is home to eight of ten states with the highest rates of new HIV diagnoses and AIDS (10). The Southeast alone (Alabama, Florida, Georgia, Kentucky, Mississippi, North Caroline, South Carolina Tennessee – Department of Health and Human Services (DHHS) Region IV) contains six of the most highly impacted states (10), with Florida, Georgia, North Carolina, and Tennessee containing 13 of the 48 counties identified as high priority for the Ending the HIV Epidemic initiative (34). The study was conducted in nineteen HIV service or research settings in four Ending the HIV Epidemic (35) priority counties that make up the Atlanta metropolitan area (Fulton, Dekalb, Cobb, and Gwinnett): two Ryan White-funded clinics (RWCs) that combined serve more than 8,000 PLWH and affiliated hospital system; eight independent RWCs; eight AIDS service organizations (ASOs); and one community-based clinical research site. These study settings were selected to enable recruitment of individuals who were retained and not retained in HIV care, recognizing the breadth and magnitude of violence experiences would likely differ between these groups.

### 2.3 Data collection

Participants completed a one-time interviewer-administered survey, provided a blood sample for HIV viral load, and completed a Release of Information form and Health Insurance Portability and Accountability Act (HIPAA) authorization form for review of medical records. Study tools and protocols were reviewed by and edited to include feedback from two Ryan White CABs and ASO board members to increase acceptability of the study.

All participants provided written informed consent prior to their participation. Surveys were administered by study staff trained in data collection and trauma-informed research methods (36–38) in a private one-on-one setting and lasted approximately 60 min. Surveys were programed in REDCap and included questions about interpersonal and community violence experiences, mental health and substance use, resilience, quality of life, HIV history, and demographics. English and Spanish versions of the survey were available, and research staff were fluent in both languages. Significant efforts were made by study staff to ensure that privacy was maintained during the interviews. Participants were informed during the consent process that if privacy were disrupted during the interview, the research staff would pause the interview and switch subjects to avoid disclosure of the study’s focus. Staff ensured the participants’ understanding of the consent process and informed them that they could pause or exit the survey at any time if they wished. Recognizing the sensitivity of the information requested and in line with trauma-informed research methods, study staff spent significant time establishing rapport with participants prior to survey administration. Upon completion, all participants (regardless of violence disclosure) received a resource guide on community-located trauma support services that was concealed in a list of other social and community services to ensure they had access to potentially helpful resources. Further, all participants received $75 USD in cash for participation.

### 2.4 Research ethics

The development of the study protocol and team training were informed by the World Health Organization ethical and safety recommendations for domestic violence research (39). The study team underwent research ethics training, as well as training trauma-informed research methods, which emphasized the importance of establishing rapport, ensuring privacy/confidentiality of study data, noting signs of adverse emotional reactions, and methods for offering and facilitating referral to mental health or other trauma support services. Strict procedures were followed to minimize loss of confidentiality. All survey data were coded with a study ID number but otherwise de-identified and consent forms with participant identifying data as well as the master list linking study ID numbers to participant names were both kept in a locked filing cabinet in a secured office, separate from the de-identified study data. All study procedures were approved by the Emory Institutional Review Board (IRB00117548). Additionally, the team consulted two Ryan White CABs to determine equitable participant compensation. Finally, recognizing the potential emotional toll of these research activities on study team members, all data collectors were provided with resources on trauma and mental health support services available to them through the university. Staff were also asked to participate in weekly team debriefing sessions, encouraged to take time off from the study as needed.

### 2.5 Eligibility and recruitment

To be eligible for the study, participants needed to be living with HIV, age ≥ 18 years, have capacity to consent, and speak either English or Spanish fluently. Purposive sampling was employed to reach PLWH across gender, race/ethnicity, and HIV care retention status (retained vs. out of care (OOC)). Participants at outpatient clinics were recruited passively through flyers and word-of-mouth and actively through direct in-person contact via a recruitment table. Participants were recruited from the ASOs through flyers and word-of-mouth. Recruitment efforts in both of these settings were bolstered through Ryan White community advisory board (CAB) and ASO board support and dissemination efforts, including troubleshooting recruitment efforts. Participants in the hospital setting were identified through examination of inpatient social worker lists of admitted PLWH. Once a potentially eligible participant was noted, the primary medical team was contacted to assess whether the patient was medically stable and had capacity to participate in a one-hour interview, and to confirm the patient was not on COVID-19 isolation precautions. These efforts were supplemented by identification of potential participants through registries of PLWH who had previously expressed interest in research participation. These individuals were contacted by email and/or phone to assess interest and eligibility for enrollment.

### 2.6 Measures

#### 2.6.1 Participant characteristics

Participants were asked to report sex assigned at birth, current gender identification, sexual orientation, race, ethnicity, highest level of educational attainment, employment status, annual household income, marital status and past-year relationship status. Additionally, participants responded to questions about the year they received an HIV diagnosis, how long ago they began receiving HIV care, whether they currently have a clinic where they receive HIV care, and if so, how long they have received care at that location. Finally, viral load was collected to assess level of viral suppression and chart data was extracted to assess retention in care to further characterize the sample.

#### 2.6.2 Violence exposure

Exposure to ACEs was captured using the Childhood Trauma Questionnaire- Short Form (CTQ-SF) (40), a 28-item measure with response options on a five-point scale from “Never True” to “Very Often True.” The CTQ-SF captures two domains of ACEs- neglect and abuse- with neglect comprised of two subtypes (emotional and physical) and abuse comprised of three subtypes (emotional, physical, and sexual). Each ACE subtype is measured using five questions (25 questions total across all subtypes), and three additional questions capture minimization and/or denial of ACEs. Dichotomous variables were created for any ACE, each ACE domain (neglect and abuse), and each ACE subtype (emotional and physical neglect and emotional, physical, and sexual abuse). For each variable, a participant was coded as “1” for lifetime ACE if they responded “yes” to any question within the overall scale, domain sub-scale, or subtype sub-scale, respectively. Items capturing minimization/denial of ACEs were not used for this analysis.

Experience of IPV was measured using the Revised Conflict Tactics Scale (CTS2) (41), a 39-item scale that captures psychological (8 items) and physical attacks (12 items), sexual coercion (7 items), physical injury (6 items), and use of negotiation (6 items) in intimate partnerships. For each prompt, participants were asked to indicate frequency of occurrence, with response options including “This has never happened,” past-year occurrence of “once,” “twice,” “3–5 times,” “6–0 times,” “11–20 times,” or “more than twenty times,” or, “not in the past year, but it did happen before.” Dichotomous variables were created for each IPV subtype (psychological, physical, sexual, injury), with each variable coded as “1” if a respondent indicated ever-experience of at least one subscale item. In a separate variable, if participants indicated any IPV experience during the past year, they were coded as “1” for past-year IPV. Items capturing negotiation were not used in the current analysis.

Non-partner violence (NPV) was captured using items from the Trauma History Questionnaire (THQ) (42), a 24-item measure of traumatic lifetime events. For each event, participants are asked to indicate if they have experienced it (yes/no), the approximate number of times they have experienced it, the approximate ages as which they experienced it, and if applicable, the participant’s relationship to the perpetrator. *Crime* was measured using four items, including having someone “take something directed from you by using force or threat of force,” “attempt to rob you or actually rob you,” or “attempt to or access in breaking into your home when you were (not there/there).” If a participant indicated “yes” to any of these four items, they were coded as having experienced crime-related NPV. S*exual violence* was measured using three items; if a participant indicated prior experience of any of these items, they were classified as having experience of sexual violence. The items included being made to “have intercourse or oral or anal sex against your will,” having someone who “touched private parts of your body, or made you touch theirs, under force or threat,” and “situations in which another person tried to force you to have unwanted sexual contact” other than that which was captured by the previous two items. Each of these items was also considered as independent dichotomous variables of “forced sex,” “unwanted physical touch,” and “other sexual violence.” Three types of physical attacks were captured, each measured dichotomously with one item: ever being attacked by someone (including family or friends) with a gun, knife, or other weapon; ever being attacked by someone (including family or friends) without a weapon; ever being beaten, spanked, or pushed by a family member hard enough to cause injury. These three questions were also aggregated into a single dichotomous variable indicating ever experience of physical violence by a family or non-family member.

Finally, experience of hate crimes was measured using an adapted version of the Anti-Gay Violence and Victimization scale, modified to include other primary reasons for discrimination/hate crime (32, 43). Twelve prompts included experiences that “might have been motivated by prejudice by others” such as “had verbal insults directed at you,” “been chased or followed,” and “been harassed by police (without assault).” Participants indicated if they have experienced each “never,” “at least once in my lifetime,” or “in the past year.” Respondents indicating lifetime or past-year experience of any hate crime were classified as having experienced a hate crime in their lifetime; respondent indicating only past-year experience of a hate crime were classified as having experienced a past-year hate crime.

### 2.7 Statistical analysis

Univariate and bivariate analysis were conducted in SAS 9.4. Means and standard deviations are reported for normally distributed continuous variables, and median and interquartile range (IQR) are reported for non-normally distributed continuous variables. Bivariate analyses were run using chi-square tests, and Fisher’s exact test when cell sizes were below 5. Significance was set at a level of *p* < 0.05. For bivariate analyses involving current gender identification, individuals identifying as transgender women or transgender females were grouped with individuals identifying as cisgender female/women; no participants identified as transgender male/men. Due to sub-sample size limitations, individuals identifying as gender queer or gender non-conforming (n = 3) or other (n = 1) were not included in gender identity analysis. Missingness was limited in the interview data (<5%) with the exception of length of receipt of HIV care (8.8% missing). Past 24-month medical record data was available for 72.6% of participants, while past 12-month medical record data was available for 90.5% of participants. Percents represent prevalence out of those with available data.

## 3 Results

### 3.1 Participant characteristics

Participants (N = 285) were primarily assigned male at birth (69.12%) and currently identified as men (63.86%); 42% of the sample identified as gay, lesbian, queer, same gender loving, or homosexual, including approximately 60% of individuals currently identifying as men (Table 1). Participants were 90.5% Black or African American and 7.7% White, while 7.1% reported Hispanic or Latinx ethnicity. Based on sex and race/ethnicity, our sample is generally representative of the Georgia HIV epidemic. According to the Georgia Department of Public Health, among people diagnosed with HIV in Georgia in 2019, 79% were male, 71% were Black, 10% were Hispanic, and 67% were MSM (44). Almost half of participants (47.7%) had a high school degree, GED, or less, while 36.8% had completed at least some college, technical, or vocational school (84.56%). Most participants were unemployed (66.7%), while those who were employed had an annual household income less than $40,000. Most participants had never been married (72.5%), and just under half had been partnered in the past 12 months (46.7%). Participants had been diagnosed with HIV a mean of 16.4 years ago (SD: 10.2) and had been engaged in care 14.2 years (SD: 9.1); of 96.5% with a current HIV care clinic, median time since they began receiving services at that clinic was 2 years (IQR:1). Most participants had been engaged in HIV care in the past 6 months (82.6%), but notably fewer had been engaged in care regularly over the past 24 months (44.2%). Eighty-six percent of the sample had been virally suppressed at some point during the 24 months prior to study enrollment, while 69.4% had been virally suppressed for the entire 24 months prior to enrollment. Similarly, just under 90% of participants had been virally suppressed at any point during the past 12 months, and 72.3% have been continuously virally suppressed.

**Table 1:** Characteristics of PLHW, Atlanta, GA, 2021–2022 (*N* = 285).

Age, median (IQR)	50 (22)	
Sex Assigned at Birth	Male	197 (69.12)
Gender, *n* (%)	Woman	90 (31.58)
	Man	182 (63.86)
	Transgender female/woman	9 (3.16)
	Transgender male/man	0
	Genderqueer or Gender Non-Conforming	3 (1.05)
	Other	1 (0.35)
Race, *n* (%)	American Indian or Alaskan Native	1 (0.35)
	Asian	0
	Black or African American	258 (90.53)
	Native Hawaiian or Pacific Islander	0
	White	22 (7.72)
	Multiracial	3 (1.05)
	Other	1 (0.35)
Ethnicity, *n* (%)	Hispanic/Latinx	20 (7.09)
Sexual Orientation, *n* (%)	Bisexual	32 (11.23)
	Gay, Lesbian, Queer, Same Gender Loving, or Homosexual	120 (42.11)
	Straight/Heterosexual	127 (44.56)
	Other	6 (2.11)
Educational Attainment, *n* (%)	High School Diploma, GED, or Less	136 (47.72)
	Some College, Technical school, or Vocational School	105 (36.84)
	4 Years of College or More	44 (15.44)
Employment Status, *n* (%)	Employed	95 (33.33)
Annual Household Income among Employed Participants (*n* = 95), *n* (%)	Less than $10,000	10 (10.64)
	$10,000–$19,999	15 (15.96)
	$20,000–$39,999	41 (43.62)
	$40,000–$59,999	17 (18.03)
	$60,000 or more	11 (11.70)
Marital status, *n* (%)	Single, never married	206 (72.54)
	Married or domestic partnership	30 (10.56)
	Widowed	11 (3.87)
	Divorced	37 (13.03)
Relationship status, *n* (%)	Partnered in Past 12 Months	133 (46.67)
Years since HIV Diagnosis, mean (SD)	16.38 (10.18)	
Years of HIV Care Engagement, mean (SD)	14.23 (9.10)	
Current HIV Care Clinic, *n* (%)	Yes	274 (96.48)
Years of Engagement with Current Clinic, median (IQR)^	2 (1)	
Retention in HIV Care, past 24 months	119 (44.24)	
Engagement in HIV Care, past 6 months	223 (82.59)	
Ever Virally Suppressed, past 24 months	237 (86.81)	
Durable Viral Suppression, past 24 months	145 (69.38)	
Ever Virally Suppressed, past 12 months	253 (89.61)	
Durable Viral Suppression, past 12 months	188 (72.31)	

^Of those with current HIV care clinic (*n* = 274).

### 3.2 Violence exposure

Prevalence of previous experiences of violence among the sample was variable by type of violence. All participants had experienced at least one adverse childhood experience (ACE), including at least one type of neglect (emotional or physical), while 93.8% had experienced some type of abuse (emotional, physical, or sexual) (Table 2). Almost 90% of participants had experienced lifetime intimate partner violence (IPV, 88.7%) or past-year IPV (87.4%, among those with past-year partnerships); experiences of IPV were largely similar between male and female participants. However, lifetime IPV resulting in injury was significantly higher among males when compared to females (38.8% vs. 20.8%, *p* = 0.0331). Ninety-seven percent of participants had experienced non-partner violence, with males assigned at birth reporting significantly higher prevalence of crime than females (81.7% vs. 53.4%, *p* < 0.001), and females reporting significantly higher prevalence of non-partner forced sex (50.0% vs. 33.5%, *p* = 0.0084). Males also reported higher lifetime and past year prevalence of hate crimes compared to women (96.9% vs. 85.2%, *p* = 0.0003, and 49.7% vs. 33.0% *p* = 0.0086, respectively). In analyses conducted by how participants currently identified their gender (Table 3) results were generally similar; all significant associations by sex assigned at birth retained significance, with the exception of IPV causing injury.

**Table 2:** Violence Exposure among PLWH, All and by Sex Assigned at Birth, Atlanta, GA, 2021–2022 (*N* = 285).

		All, N (%) *N* = 285	Sex Assigned at Birth, *N* (%) *N* = 285		
			Male n = 197	Female n = 88	*p*
Any Adverse Childhood Experience (ACE)		269 (100.00)	187 (100.00)	82 (100.00)	–
	Any ACE, Subtype: Neglect	279 (100.00)	194 (100.00)	85 (100.00)	–
	Emotional Neglect	279 (100.00)	194 (100.00)	85 (100.00)	–
	Physical Neglect	284 (100.00)	196 (100.00)	88 (100.00)	–
	Any ACE, Subtype: Abuse	257 (93.80)	179 (94.71)	78 (91.76)	0.3500
	Emotional Abuse	226 (80.43)	160 (82.47)	66 (75.86)	0.1965
	Physical Abuse	241 (85.77)	170 (87.63)	71 (81.61)	0.1818
	Sexual Abuse	185 (66.07)	126 (65.28)	59 (67.82)	0.6789
Any Lifetime Intimate Partner Violence (IPV)*		118 (88.72)	78 (91.76)	40 (83.33)	0.1399
	Psychological IPV	116 (87.22)	76 (89.41)	40 (83.33)	0.3133
	Physical Assault IPV	64 (48.12)	45 (52.94)	19 (39.58)	0.1387
	Sexual IPV	46 (34.59)	33 (38.82)	13 (27.08)	0.1716
	Injury IPV	43 (32.33)	**33 (38.82)**	**10 (20.83)**	**0.0331**
Any Past-Year Intimate Partner Violence (IPV)*		111 (87.40)	74 (90.24)	37 (82.22)	0.1926
Psychological IPV		111 (86.05)	73 (87.95)	38 (82.61)	0.4015
Physical Assault IPV		55 (41.35)	38 (44.71)	17 (35.42)	0.2961
Sexual IPV		40 (30.30)	29 (34.12)	11 (23.40)	0.1997
Injury IPV		34 (25.76)	26 (30.95)	8 (16.67)	0.0710
Any Non-Partner Violence (NPV)		267 (97.45)	184 (97.87)	83 (96.51)	0.6816
NPV: Crime		208 (72.98)	**161 (81.73)**	**47 (53.41)**	**<0.001**
NPV: Sexual Violence		192 (68.82)	132 (69.11)	60 (68.18)	0.8764
NPV: Forced Sex		109 (38.65)	**65 (33.51)**	**44 (50.00)**	**0.0084**
NPV: Unwanted Physical Touch		90 (32.03)	62 (32.12)	28 (31.82)	0.9593
NPV: Other Sexual Violence		32 (11.43)	22 (11.46)	10 (11.36)	0.9816
NPV: Physical Attack		111 (39.22)	70 (35.90)	41 (46.59)	0.0881
NPV: Physical Attack with a Weapon		100 (35.34)	70 (35.90)	30 (34.09)	0.7685
NPV: Physical Attack without a Weapon		54 (19.08)	39 (20.00)	15 (17.05)	0.5582
NPV: Family-Perpetuated Injury		63 (22.26)	48 (24.62)	15 (17.05)	0.1565
Ever Experience of a Hate Crime		262 (93.24)	**187 (96.89)**	**75 (85.23)**	**0.0003**
Past-Year Experience of a Hate Crime		125 (44.48)	**96 (49.74)**	**29 (32.95)**	**0.0086**

*Among partnered individuals, *n* = 127.

**Table 3:** Violence Exposure by Gender Identity among PLWH, Atlanta, GA, 2021–2022 (*N* = 281).

			Gender, *N* (%)		
			Man *N* = 182	Cis/Trans Woman *N* = 99	*p*
Any Adverse Childhood Experience (ACE)			173 (100.00)	93 (100.00)	–
	Any ACE, Subtype: Neglect		179 (100.00)	96 (100.00)	–
		Emotional Neglect	179 (100.00)	96 (100.00)	–
		Physical Neglect	181 (100.00)	99 (100.00)	–
	Any ACE, Subtype: Abuse		166 (94.86)	88 (91.67)	0.3002
		Emotional Abuse	148 (82.68)	74 (76.51)	0.1525
		Physical Abuse	159 (88.83)	79 (80.91)	0.0602
		Sexual Abuse	117 (65.36)	66 (67.35)	0.7388
Any Lifetime Intimate Partner Violence (IPV)*			70 (92.11)	46 (83.64)	0.1330
	Psychological IPV		68 (89.47)	46 (83.64)	0.3265
	Assault IPV		40 (52.63)	23 (41.82)	0.2215
	Sexual IPV		28 (36.84)	17 (30.91)	0.4804
	Injury IPV		27 (35.53)	14 (25.45)	0.2199
Any Past-Year Intimate Partner Violence (IPV)*			67 (90.54)	42 (82.35)	0.1781
	Psychological IPV		65 (87.84)	44 (83.02)	0.4426
	Assault IPV		33 (43.42)	21 (38.18)	0.5477
	Sexual IPV		25 (32.89)	14 (25.93)	0.3929
	Injury IPV		21 (27.63)	11 (20.37)	0.3436
Any Non-Partner Violence (NPV)			169 (97.69)	94 (96.91)	0.7044
	NPV: Crime		**148 (81.32)**	**56 (56.57)**	**<0.001**
	NPV: General Violence		169 (94.94)	92 (94.85)	0.9717
	NPV: Sexual Violence		122 (69.32)	68 (68.69)	0.9134
	NPV: Forced Sex		**59 (32.96)**	**49 (49.49)**	**0.0068**
	NPV: Unwanted Physical Touch		56 (31.46)	34 (34.34)	0.6235
	NPV: Other Sexual Violence		17 (9.60)	14 (14.14)	0.2523
	NPV: Physical Attack		66 (36.67)	44 (44.44)	0.2034
	NPV: Physical Attack with a Weapon		66 (36.67)	33 (33.33)	0.5777
	NPV: Physical Attack without a Weapon		36 (20.00)	18 (18.18)	0.7130
	NPV: Familial Injury		46 (25.56)	17 (17.17)	0.1090
Ever Experience of a Hate Crime			**173 (97.19)**	**85 (85.86)**	**0.0003**
Past-Year Experience of a Hate Crime			**89 (50.00)**	**33 (33.33)**	**0.0074**

*Among partnered individuals, *n* = 127. The bold indicated where there were significant statistical differences between groups.

Among participants currently identifying as men, several significant differences in violence experiences were apparent by sexual orientation (Table 4). Prevalence of childhood emotional, physical, and sexual abuse varied significantly across sexual orientation, with gay men reporting the highest prevalence of each emotional (87.1%), physical (93.6%), and sexual abuse (74.3%), followed by bisexual men (84.6, 84.6, and 65.4%) and heterosexual men (68.3, 78.1, 39.0%, *p* = 0.0300, *p* = 0.0200, and *p* = 0.0003, respectively). Similar relationships were seen across non-partner forced sex, with 40.0% of gay, 36.0% of bisexual, and 12.2% of heterosexual men reporting previous experiences (*p* = 0.0051). By contrast, bisexual men were most likely to report previous experience of physical attack without a weapon (44.0%) or physical attack by a family member resulting in injury (32.0%) compared to gay men (18.0 and 28.8%) and heterosexual men (9.8% for both, *p* = 0.0287 and *p* = 0.0040, respectively). Finally, bisexual men were least likely to report a lifetime hate crime experience (88.5%) compared to heterosexual (95.1%) and gay men (100.0%, *p* = 0.0040).

**Table 4:** Violence Exposure by Sexual Identity among Self-Identifying Men Living with HIV, Atlanta, GA, 2021–2022 (*N* = 179).

		Sexual Identity, N (%)			
		Bisexual n = 26	Gay n = 112	Heterosexual n = 41	p
Any Adverse Childhood Experience (ACE)		25 (100.00)	105 (100.00)	40 (100.00)	–
	Any ACE, Subtype: Neglect	25 (100.00)	111 (100.00)	40 (100.00)	–
	Emotional Neglect	25 (100.00)	111 (100.00)	40 (100.00)	–
	Physical Neglect	26 (100.00)	111 (100.00)	41 (100.00)	–
	Any ACE, Subtype: Abuse	24 (92.31)	102 (97.14)	37 (90.24)	0.1351
	Emotional Abuse	**22 (84.62)**	**95 (87.16)**	**28 (68.29)**	**0.0300**
	Physical Abuse	**22 (84.62)**	**102 (93.58)**	**32 (78.05)**	**0.0200**
	Sexual Abuse	**17 (65.38)**	**81 (74.31)**	**16 (39.02)**	**0.0003**
Any Lifetime Intimate Partner Violence (IPV)*		13 (100.00)	41 (89.13)	15 (93.75)	0.7138
	Psychological IPV	13 (100.00)	40 (86.96)	14 (87.50)	0.4954
	Assault IPV	9 (69.23)	24 (52.17)	6 (37.50)	0.2367
	Sexual IPV	7 (53.85)	16 (32.61)	5 (31.25)	0.3613
	Injury IPV	7 (53.85)	16 (34.78)	4 (25.00)	0.3026
Any Past-Year Intimate Partner Violence (IPV)*		13 (100.00)	38 (86.36)	15 (93.75)	0.4713
	Psychological IPV	13 (100.00)	37 (84.09)	14 (87.50)	0.3673
	Assault IPV	5 (38.46)	21 (45.65)	6 (37.50)	0.8041
	Sexual IPV	7 (53.85)	13 (28.26)	4 (25.00)	0.1732
	Injury IPV	4 (30.77)	13 (28.26)	4 (25.00)	1.0000
Any Non-Partner Violence (NPV)		25 (100.00)	103 (97.17)	38 (97.44)	1.0000
	NPV: Crime	23 (88.46)	92 (82.14)	30 (73.17)	0.3036
	NPV: General Violence	26 (100)	102 (93.58)	38 (95.00)	0.5409
	NPV: Sexual Violence	17 (68.00)	81 (74.31)	21 (53.85)	0.0605
	NPV: Forced Sex	**9 (36.00)**	**44 (40.00)**	**5 (12.20)**	**0.0051**
	NPV: Unwanted Physical Touch	10 (40.00)	38 (34.55)	7 (17.50)	0.0842
	NPV: Other Sexual Violence	4 (16.00)	11 (10.09)	2 (5.00)	0.3380
	NPV: Physical Attack	13 (52.00)	37 (33.33)	15 (36.59)	0.2165
	NPV: Physical Attack with a Weapon	13 (52.00)	37 (33.33)	15 (36.59)	0.2165
	NPV: Physical Attack without a Weapon	**11 (44.00)**	**20 (18.02)**	**4 (9.76)**	**0.0043**
	NPV: Familial Injury	**8 (32.00)**	**32 (28.83)**	**4 (9.76)**	**0.0287**
Ever Experience of a Hate Crime		**23 (88.46)**	**108 (100.00)**	**39 (95.12)**	**0.0040**
Past-Year Experience of a Hate Crime		17 (65.38)	49 (45.37)	20 (48.78)	0.1862

*Among partnered individuals, *n* = 127. The bold indicated where there were significant statistical differences between groups.

## 4 Discussion

Over the past two decades, the link between experience of violence and poor HIV outcomes has been well-established (21, 23–25), with numerous national advisory groups and agencies calling for integration of violence screening and support within HIV services (45–47). The bulk of this literature has been in cis-gender women and has been limited to exploration of IPV and childhood abuse histories (11–13, 15–17, 30, 31, 48). The present study is the first study to our knowledge to comprehensively explore interpersonal and community forms of violence among PLWH across gender and sexual orientation, including both those retained and out of HIV care, in one sample. In doing so, it elucidates the many forms of violence health systems and ASOs should be equipped to screen and provide support for, and further emphasizes the importance of integrating trauma-informed care in these settings.

The experience of the various forms of violence reported in our study is similar and/or higher to other studies of PLWH. For example, 100% of our sample reported at least one ACE and nearly all (94%) reported experiencing physical, sexual, and/or emotional abuse as a child. In a study of 584 PLWH at risk for alcohol use disorders, the majority of whom were white men, 83% reported experience of at least one ACE and reporting of childhood abuse was lower than our sample (i.e., 46% vs. 80% for emotional abuse; 34% vs. 86% for physical abuse; 26 vs. 66% for sexual abuse) (31). A recent systematic review of global studies exploring impact of childhood sexual violence on antiretroviral therapy adherence, found reporting of childhood sexual violence ranged from 7–55% (30). Although tools used to assess violence experience differed, frequencies were substantially lower than that reported in our sample (30). In our sample, nine in ten reported lifetime and past-year IPV. These frequencies are also higher than estimates provided by a recent meta-analysis of 49 studies worldwide examining IPV among PLWH (39% vs. 89% any IPV; 28% vs. 86% emotional IPV; 26% vs. 41% physical IPV, and17% vs. 30% sexual IPV) (48). Experience of NPV in our sample is similar to other studies of PLWH. Specifically, over 90% of females in our sample reported experiencing NPV of some type, which is slightly higher, but in line with a sample of Canadian women with HIV reporting 81% experiencing NPV in their lifetime (49). It should be noted that the literature is very limited on NPV among PLWH, as most studies do not provide violence data by perpetrator, thus our findings add substantially to the field. Finally, experience of hate crimes among our sample was high (93% reported lifetime experience of a hate crime; 44% reported a past year experience of a hate crime), which is higher than reported in other studies of PLWH, including one study among transgender women reported 46% had experienced a transphobic hate crime, although this study only examined experiences of hate crimes related to being transgender and not other aspects of their identity such as race/ethnicity (50). Though we cannot ascertain why there are similarities and differences between our sample and others reported in the literature across an array of violence exposures, it is abundantly clear that in studies among PLWH, including ours, violence experiences are excessively common among this population.

Our study further expands the literature on violence experienced by PLWH by including individuals assigned male and female at birth in a single sample, thus allowing us to examine differences in violence experiences by sex. Overall, violence was ubiquitous and very few statistically significant differences were identified, with two notable exceptions. Crime (of various forms, including hate crimes) was experienced significantly more among individuals assigned male at birth than female at birth, and non-partner sexual violence was experienced more among individuals assigned female at birth than male at birth. Notably, rates of IPV were similarly high across males and females, with males reporting more IPV-associated injury requiring treatment than females.

National healthcare organizations including Health Resources and Services Administration (HRSA) have put forth recommendations for integrating IPV screening and intervention within healthcare settings, and the Affordable Care Act includes screening and brief counseling for IPV as part of required free preventive services for women. In support of the recommendation, HRSA cites the efficacy of IPV screening alongside provision of education about support services in enhancing support service utilization, mental and physical health, and safety, and reducing incident IPV (51, 52). Additionally, the HIV Primary Care Guidelines of the Infectious Diseases Society of America now recommend screening for IPV at initial evaluation and “periodic intervals” thereafter (46), and the US Preventive Services Task Force recommends IPV screening of women of reproductive age with provision/referral to support services (53). However, as our data supports, IPV is experienced frequently by males as well and thus IPV screening should be recommended and conducted among all PLWH, not just women. Further, IPV is only one dimension of the total violence experienced by PLWH, and solely focusing on IPV screening and intervention may miss opportunities to address other forms of violence that could be determinantal to the wellness of PLWH.

The near universal experience of multiple forms of violence by our entire sample validates recent calls for HIV care settings to provide trauma-informed care. Specifically, the Health Resources and Services Administration Ryan White HIV/AIDS Program and National Alliance of State and Territorial AIDS Directors have called for integration of trauma-informed care into HIV services (45, 54). Trauma-informed care is defined by the Substance Abuse and Mental Health Service Administration as an organizational approach that “realizes the widespread impact of trauma and understands potential paths for recovery; recognizes the signs and symptoms of trauma in clients, families, staff and others involved with the system; and responds by fully integrating knowledge about trauma into policies, procedures, and practices and seeks to actively resist re-traumatization” (55). Based on the literature on which our study builds, coupled with our findings, trauma-informed care is urgently needed within all setting serving PLWH in Atlanta, and likely across the Southern US and beyond.

Our study findings must be interpreted in the context of the strengths and limitations of the study. Key study strengths include rigorous staff training on methods to establish interviewer-participant rapport, ensure interview privacy, and data confidentiality to promote participant safety as well as the validity of data captured. Additional strengths included the comprehensiveness of violence forms examined using validated instruments, inclusion of PLWH across gender, sexual minorities, and racial/ethnic minorities, and diversity of types of study settings from which participants were recruited (i.e., clinics, ASOs, and hospitals) to yield a diverse sample of PLWH – recognizing the forms, frequency and severity of violence experienced may vary by those who were well-retained in care versus out-of-care. Key study limitations are the low number of PLWH who were out of care in spite recruitment from ASO and hospital-based settings, who we expect would report higher levels and forms of violence experience than reported here, and the reliance on self-reported data only, which could introduce recall bias.

In conclusion, among our sample of PLWH at the epicenter of the US HIV epidemic, histories of interpersonal and community violence are common. Prior research from other samples linking IPV and childhood abuse to poor HIV (22–25) outcomes suggest the high levels of violence reported in the present study may help explain the significant shortcomings along the HIV care continuum in the US South. Our study findings emphasize the need for RWCs, ASOs, and hospital systems to be universally trained in trauma-informed approaches and have integrated onsite mental health and social support services. To help prioritize violence screening and support resources, our team will next be examining which forms of violence have greatest impact on retention in HIV care and viral suppression. However, the high levels of some forms of violence (i.e., childhood neglect, IPV), suggest extensive violence screening may in fact not be necessary but rather a universal trauma-informed approach should be employed with all patients and screening resources should be dedicated to assessment of danger or potential mediators in the violence to HIV outcome pathway (i.e., PTSD and/or substance abuse).

## Data availability statement

The datasets presented in this article are not readily available because the study team is still actively analyzing this data. After we analyze the data for the primary purposed of our study, the analyzed dataset will be available upon reasonable request to the corresponding author. Requests to access the datasets should be directed to JS, jmcderm@emory.edu.

## Ethics statement

The studies involving humans were approved by Emory University Institutional Review Board. The studies were conducted in accordance with the local legislation and institutional requirements. The participants provided their written informed consent to participate in this study.

## Author contributions

JS: Conceptualization, Funding acquisition, Project administration, Resources, Supervision, Writing – original draft, Writing – review & editing. KA: Formal Analysis, Writing – original draft, Writing – review & editing. ML: Methodology, Supervision, Writing – review & editing. SG: Writing – original draft, Writing – review & editing. SH: Data curation, Project administration, Writing – original draft. ER: Project administration, Supervision, Writing – review & editing. MC: Writing – original draft, Writing – review & editing. AK: Conceptualization, Data curation, Funding acquisition, Supervision, Writing – original draft, Writing – review & editing.
